# Beta-defensins and analogs in *Helicobacter pylori* infections: mRNA expression levels, DNA methylation, and antibacterial activity

**DOI:** 10.1371/journal.pone.0222295

**Published:** 2019-09-19

**Authors:** Raffaela Pero, Tiziana Angrisano, Mariarita Brancaccio, Annarita Falanga, Lucia Lombardi, Francesco Natale, Sonia Laneri, Barbara Lombardo, Stefania Galdiero, Olga Scudiero

**Affiliations:** 1 Dipartimento di Medicina Molecolare e Biotecnologie Mediche, Università degli Studi di Napoli “Federico II”, Napoli, Italy; 2 Task Force sugli Studi del Microbioma, Università degli Studi di Napoli “Federico II”, Napoli, Italy; 3 Dipartimento di Biologia, Università degli Studi di Napoli “Federico II”, Napoli, Italy; 4 Dipartimento di Biologia ed Evoluzione degli Organismi Marini, Stazione Zoologica Anton Dohrn, Napoli, Italy; 5 Dipartimento di Farmacia, Università degli Studi di Napoli “Federico II”, Napoli, Italy; 6 Dipartimento di Agraria, Università degli Studi di Napoli “Federico II”, Napoli, Italy; 7 CEINGE-Biotecnologie Avanzate Scarl, Napoli, Italy; Oita University Faculty of Medicine, JAPAN

## Abstract

Antimicrobial peptides can protect the gastric mucosa from bacteria, but *Helicobacter pylori (H*. *pylori)* can equally colonize the gastric apparatus. To understand beta-defensin function in *H*. *pylori*-associated chronic gastritis, we investigated susceptibility, human beta-defensin mRNA expression, and DNA methylation changes to promoters in the gastric mucosa with or without *H*. *pylori* infection. We studied the expression of HBD2 (gene name *DEFB4A*), HBD3 (*DEFB103A*), and HBD4 (*DEFB104*) using real-time PCR in 15 control and 10 *H*. *pylori* infection patient gastric specimens. This study demonstrates that *H*. *pylori* infection is related to gastric enhancement of inducible HBD2, but inducible HBD3 and HBD4 expression levels remained unchanged. HBD2 gene methylation levels were overall higher in *H*. *pylori*-negative samples than in *H*. *pylori*-positive samples. We also assessed antimicrobial susceptibility using growth on blood agar. The *H*. *pylori strain Tox+* was susceptible to all defensins tested and their analogs (3N, 3NI). These results show that HBD2 is involved in gastritis development driven by *H*. *pylori*, which facilitates the creation of an epigenetic field during *H*. *pylori-*associated gastric tumorigenesis.

## Introduction

Chronic inflammation in specific organs is associated with increased cancer risk, including ulcerative colitis (UC)-associated colon cancer, liver cancer, and gastric cancer. Storage of epigenetic modifications caused by chronic inflammation seems to correlate with tumor progression [1–6]. Chronic inflammation is correlated with the incidence of cancer development [7–9]. Chronic inflammation is triggered by infection of *Helicobacter pylori (H*. *pylori)* in the stomach, and it causes a predisposition in gastric cancer development [10,11].

DNA methylation is involved in chronic inflammation-mediated carcinogenesis [12,13]. Niwa et al. shown that DNA methylation modifications in gastrointestinal mucosae subsequent to *H*. *pylori* infection can be transient or permanent, and it is the infection-associated inflammatory response rather than *H*. *pylori* itself that can cause DNA methylation [14].

Previous studies demonstrated that [15,16] DNA methylation modifications are present in *H*. *pylori*-infected stomachs, in livers infected with viruses, or in the rectum or colon in the case of UC [17,18]. DNA methylation alterations in non-cancerous inflammatory tissues represent one of the preliminary stages of tumor transformation [19]. Gastritis activity is linked to the evolution of widespread stomach cancer [20]. Many onco-suppressors were detected both in gastric cancer and chronic gastritis [21]. Inactivation of p53, E-cadherin, hMSH2, hMLH1, and microsatellite instability (MSI) are well-recognized examples. However, disparate studies have showed that mutation or deletion is an uncommon process of inactivating these well-established suppressor genes. DNA methylation represents a key mechanism for suppressor gene inactivation [22] and a critical element for the early events of gastric carcinogenesis [23]. Methylation of the E-cadherin DNA promoter is related to *H*. *pylori* status [24] independent of gastritis type and patient age.

Also, antibiotic eradication of *H*. *pylori* infection reverses DNA methylation of the E-cadherin promoter [24]. Similarly, the analysis of *H*. *pylori’s* effect on DNA methylation of different genes (HAND-1, p16, LOX, HRASLS, THBD, and P41ARC) in healthy patients with or without *H*. *pylori* infection and patients affected by gastric cancer revealed that DNA methylation levels were higher in patients with *H*. *pylori* infection compared to healthy patients. These data suggest that *H*. *pylori* infection causes DNA methylation especially in premalignant lesions rather than gastric cancer [15, 25].

Antimicrobial peptides (AMPs) are significantly involved in native immune reactions against diverse microbes, and they constitute a promising substitute for antibiotics to resolve the problem of microbial resistance [26]. Defensins are a remarkable set of antimicrobial peptides. They are cationic peptides that have similar folding and an identical six-cysteine motif to disulfide bonds. They are synthesized either constitutively (HBD1) or inducibly by microbe components or inflammation (HBD2, HBD3, HBD4) [26].

Boughan et al., [27] found that gene expression of HBD2 demanded *cag*PAI and was NOD1-dependent. In contrast, HBD3 expression was NOD1-independent but relied on epidermal growth factor receptor (EGFR)-mediated ERK activation. In contrast to a study in AGS cells [27] that established the involvement of NOD1 in HBD2 expression only, other data suggest that NOD1 mediates both HBD2 and HBD3 expression in HEK293 cells in the presence of *H*. *pylori* [28]. Both HBD2 and HBD3 are differentially present in gastric mucosa in connection to *H*. *pylori* status, and *H*. *pylori* infection is related to hBD3 expression reduction in chronic gastritis [29]. In addition, HBD3 release from *H*. *pylori*-infected gastric epithelial cells happens via a new EGFR-activating pathway during a premature step of *H*. *pylori* infection [30]. Otherwise, HBD1 is constitutively expressed in non-inflamed normal tissue [31], which calls attention to its significance in defense versus microbial infection. Decreased HBD1 expression was found in *H*. *pylori* infected humans [32]. In addition, Patel et al., [33] found that *H*. *pylori* downregulates HBD1 expression via NF-κB signaling, suggesting that this may prolong survival and persistence of bacteria in the stomach niche. These studies, although conflicting, suggest that *H*. *pylori* may regulate HBD1 expression.

Defensins exert their killing activity against bacteria, yeasts, fungi, and viruses. The activity of antimicrobial peptides involves the initial electrostatic binding to the cell membrane of microorganisms and subsequent incorporation into the membrane. This antimicrobial action induces damage or even destruction of the microbial cell membrane via pore formation [34]. The antimicrobial activity of HBD1, HBD2 and HBD4 is reduced by high NaCl concentrations, while HBD3 is the only defensin still active up to 150 mM NaCl concentrations. We previously reported the killing activity of β-defensin analogs designed from HBD1 and HBD3, showing that they have augmented potency or decreased sensitivity to high ionic strength. We examined their antibacterial, antiviral, and chemotactic actions, including salt resistance. Based on our analysis, we demonstrated that the C-terminal domain (RRKK) of HBD3 and the inner domain of HBD1 (PIFTKIQGT) are essential for antimicrobial activity. The removal of six ends at the N-terminus of HBD3 did not decrease the activity. Consequently, we developed an analog that maintained the HBD1 killing activity and was resistant to high NaCl concentrations like HBD3 [35–38]. The activity of this analog (3NI) was not evaluated against *H*. *pylori*, and it may represent a promising tool against *H*. *pylori* that can be used to combat infection and reverse DNA methylation.

Here, we hypothesized that the degree of altered DNA methylation in gastric apparatus is correlated with the activity of gastritis as methylation observed in the inflamed stomach would elicit diffuse-type cancer development. This study aimed to clarify whether DNA methylation modifications and human beta-defensins levels are related to inflammation activity in the *H*. *pylori*-infected stomach mucosa. We evaluated methylation levels of promoter CGIs of beta-defensin genes (HBD2-4) in gastric biopsy specimens from 15 controls and 10 patients colonized by *H*. *pylori*. *H*. *pylori* can cause the induction of HBD2 but several studies also report the overexpression of HBD3 [39–42], so we also evaluated the mRNA induction pattern of HBD2-4 by *H*. *pylori*. Finally, we tested the susceptibility of *H*. *pylori* to treatment by synthetic ß-defensin analogs.

## Materials and methods

### Tissue sample

The protocol was created based on the Declaration of Helsinki and approved by the Ethics Committee of the University of Naples “Federico II”. Appropriate written informed consent was collected before all procedures.

Non-tumoral mucosa specimens with or without chronic gastritis were acquired from 25 patients recruited at the University of Naples “Federico II”, Italy. After tissue removal, all samples were immediately frozen and fixed in 100 mL/L formalin.

#### *H*. *pylori* infection diagnosis

Sections were stained with Giemsa, and the rapid urease test (CLO test) was carried out with fresh samples collected from the gastric corpus and antrum. *H*. *pylori* infection was assumed positive when *H*. *pylori* was detected or the CLO test was positive.

Patient characteristics are summarized in Table 1.

**Table 1:** Patient characteristics.

Case	Sex	Age (y)	Biopsy site	Urease activity	Severity of gastritis*	Symptom or diagnosis
**1**	F	45	Antrum	Negative	Mild	No symptom
**2**	F	46	Antrum	Negative	Normal	No symptom
**3**	F	33	Antrum	Negative	Normal	No symptom
**4**	F	37	Antrum	Negative	Normal	Epigastric discomfort
**5**	M	43	Antrum	Negative	Normal	Epigastric discomfort
**6**	M	48	Corpus	Negative	Mild	Epigastric discomfort
**7**	F	35	Antrum	Negative	Mild	Epigastric discomfort
**8**	F	41	Corpus	Negative	Mild	Chronic gastritis
**9**	F	40	Antrum	Negative	Mild	Chronic gastritis
**10**	F	37	Antrum	Negative	Normal	Chronic gastritis
**11**	F	39	Antrum	Negative	Mild	Chronic gastritis
**12**	F	45	Antrum	Negative	Mild	Chronic gastritis
**13**	F	50	Antrum	Negative	Normal	Chronic gastritis
**14**	F	33	Corpus	Negative	Mild	Chronic gastritis
**15**	F	44	Antrum	Negative	Normal	Chronic gastritis
**16**	M	42	Antrum	Positive	Moderate	Chronic gastritis
**17**	M	45	Antrum	Positive	Moderate	Chronic gastritis
**18**	M	34	Antrum	Positive	Moderate	No symptom
**19**	M	39	Corpus	Positive	Severe	No symptom
**20**	F	40	Corpus	Positive	Severe	No symptom
**21**	F	57	Antrum	Positive	Moderate	Epigastric discomfort
**22**	F	65	Antrum	Positive	Moderate	Epigastric discomfort
**23**	M	53	Antrum	Positive	Severe	Epigastric discomfort
**24**	M	66	Corpus	Positive	Severe	Epigastric discomfort
**25**	M	23	Corpus	Positive	Severe	Chronic gastritis

*According to the updated Sydney system

### Real-time qPCR analysis

Total RNA was extracted as previously stated [43]. Total RNA was reverse-transcribed with Quanti-Tect^®^ Reverse Transcription (Qiagen) using oligo-dT and random primers according to the manufacturer’s instructions as previously described [44]. Quantitative PCR was performed with Quanti-Tect SYBR Green (Qiagen) using a Chromo 4 Real-Time thermocycler (BIORAD). The following primers were used for HBD2-4 cDNA amplification: (HBD2F) 5′- ATCAGCCATCAGGGTCTTGT-3′ and (HBD2R) 5′- GAGACCACAGGTGCCAATTT-3′, HBD3fw 5′- TGAAGCCTAGCAGCTATGAGGATC-3′ and HBD3rv 5′- CCGCCTCTGACTCTGCAATAA-3′, HBD4F 5′-AGATCTTCCAGTGAGAAGCGA-3′ and HBD4R 5′-GACATTTCTTCCGGCAACGG-3′. G6PD and 18S rRNA genes were old as house-keeping genes for PCR reaction: G6F (forward) 5′-ACAGAGTGAGCCCTTCTTCAA-3′ and G6R (reverse) 5′-GGAGGCTGCATCATCGTACT-3′, and 18SF: (forward) 5′-GCGCTACACTGACTGGCTC-3′ and 18SR (reverse) 5′- CATCCAATCGGTAGTAGCGAC-3′. The quantitative PCR conditions were 95°C for 15 min, 40 cycles of 95°C for 15 s, 59°C for 30 s, and 72°C for 30 s. Calculations of relative expression levels were performed using the 2^−ΔΔCt^ method [45], taking into account at least 3 independent experiments.

### DNA methylation analysis

Genomic DNA was isolated using the DNeasy extraction kit (Qiagen) according to the manufacturer’s instructions. Sodium bisulfite conversion was performed using the EZ DNA Methylation Kit (Zymo Research). The manufacturer’s protocol was followed using 1 μg of genomic DNA with elution into 30 mL of H2O.

#### Bisulfite genomic sequencing

50 ng of each sample was used as template in PCR reactions using the following primers to analyze the HBD2 promoter region: HBD2/BsF 5′- GGAAGGATAGGGTTTTGAGAGATAT -3′ (position from nucleotides -944 to -920) and HBD2/BsR 5′-AACCAAAACTTTCTCTACTTTCCAC -3′ (nucleotides -781 to -757), HBD3/BsF 5′-GGTAGGTTTTAGATAATGATGAAG -3′ (nucleotides -732 to -709) and HBD3/BsR 5′- ACCCCCTAAATAACTAAAACC -3′ (nucleotides -518 to -498), HBD4/BsF 5′- TAGGTTAGGAGGGTTTTATGGATTT -3′ (nucleotides -1907 to -1883) and HBD4/BsR 5′- CCAACAAACATAACCCAACTCTAAT -3′ (nucleotides -1738 to -1714). The Fully Human DNA Methylated (ZYMO RESEARCH) was used as a completely methylated DNA control. Amplification was performed using Hot-Star Taq DNA polymerase (QIAGEN) under the following conditions: 15 min at 95°C, 40 cycles of 30 sec at 95°C, 30 sec at 54°C, 1 min at 72°C, and a final elongation of 10 min at 72°C before holding at 4°C in a final volume of 25 μl. PCR product quality and contamination was assessed using a 1.5% agarose gel with ethidium bromide staining. PCR products were cloned into the pGEM^®^-T Easy vector (Promega). Plasmid DNA was purified using the Qiagen Plasmid Mini Kit. Plasmids were purified and sequenced in either direction with T7 and Sp6 primers. At least 10 independent clones were sequenced to calculate the DNA methylation pattern of single molecules.

The methylation level of CG cytosines at a given position within the sequence can be calculated as percent methylation as follows: %M = [C^M^/ (C^M^ + C^U^)] × 100, where C^M^ is the number of clones with methylation at that cytosine and C^U^ is the number of clones unmethylated at that cytosine. Precision is improved by increasing the number of analyzed clones, unless there is bias in the cloning or amplification steps.

### Peptide synthesis

The peptides HBD2-4 were purchased from Peptides International, Inc.-USA. The analogs (3N and 3NI) were synthesized on a Rink Amide Resin using Fmoc. The first pairing of individual amino acids was performed in the presence of 4 equivalents of Fmoc-protected amino acid, four equivalents of OXIMA, and four equivalents of DIC. The second coupling was done with four equivalents of HATU and eight equivalents of DIPEA. The Fmoc deprotection was achieved with two cycles of a piperidine solution (30% v/v in DMF). The crude peptides were cleaved from the resins with a TFA acid solution in the presence of scavengers. RP-HPLC was used to purify the crude peptides, which were identified using LC-MS.

The sequence of the peptides used in this study is indicated in Table 2.

**Table 2:** The sequence of the peptides used in this study.

**HBD2**	**GIGDPVTC**^**1**^**LKSGAIC**^**2**^**HPVFC**^**3**^**PRRYKQIGTC**^**4**^**GLPGTKC**^**5**^**C**^**6**^**KKP**
**HBD3**	**GIINTLQKYYC**^**1**^**RVRGGRC**^**2**^**AVLSC**^**3**^**LPKEEQIGKC**^**4**^**STRGRKC**^**5**^**C**^**6**^**RRKK**
**HBD4**	**ELDRIC**^**1**^**GYGTARC**^**2**^**RKK-C**^**3**^**RSQEYRIGRC**^**4**^**PNTYAC**^**5**^**C**^**6**^**LRK**
**3N**	**QKYYC**^**1**^**RVRGGRC**^**2**^**AVLSC**^**3**^**LPKEEQIGKC**^**4**^**STRGRKC**^**5**^**C**^**6**^**RRKK**
**3NI**	**KYYC**^**1**^**RVRGGRC**^**2**^**AVLSC**^**3**^**PIFTKIQGTC**^**4**^**STRGRKC**^**5**^**C**^**6**^**RRKK**

### Antimicrobial assay

CFU assays of the antimicrobial activities of HBDs against *H*. *pylori* (ATCC49503 (tox+ strain 60190); American Type Culture Collection, ATCC) were performed. The toxin-producing *H*. *pylori* strain was revived from frozen stocks by seeding on a blood agar plate (7% horse blood) at 37°C for 3 days in microaerophilic conditions (O_2_:10% and CO:10%) generated with Anaeropack Campylo (Mitsubishi Gas Chemicals Corp.). Bacteria harvested from the plates were suspended in 200 ml of brain heart infusion broth (10% fetal calf serum) and grown in liquid culture at 37°C for three days while shaking in a monitored microaerophilic atmosphere.

*H*. *pylori* was incubated with HBDs for two hours at 37°C. Two concentrations of peptides (2.5 μM and 12.5 μM) were used. For the salt dependence assay, 0, 50, 100, and 200 mM concentrations of NaCl were added in the incubation buffer as previously described. Each assay was done in triplicate.

### Statistical analysis

Student’s t-test assessed statistical significance between groups. Data are expressed as means S ± standard deviation (SD). Each experiment was repeated at least three times. Results were evaluated to be statistically significant at a p-value < 0.01 or 0.05.

### Databases

The Ensembl database retrieved gene sequences: HBD-2, accession number ENST00000318157; HBD-3 accession number ENST0000031435; HBD4, accession number ENSG00000176782.

## Results

### HBD2 expression and DNA methylation analysis in *H*. *pylori-*positive patients

To evaluate the influence of *H*. *pylori* colonization in gastric tissues, we evaluated the *HBD2* gene expression in mucosal samples gastritis from 10 *H*. *pylori*-positive and 15 *H*. *pylori* negative patients, by qPCR analysis. *H*. *pylori* positive samples exhibited a moderate chronic active gastritis, whereas the *H*. *pylori*-negative specimens displayed no or minimal chronic inflammation.

**Fig 1:**
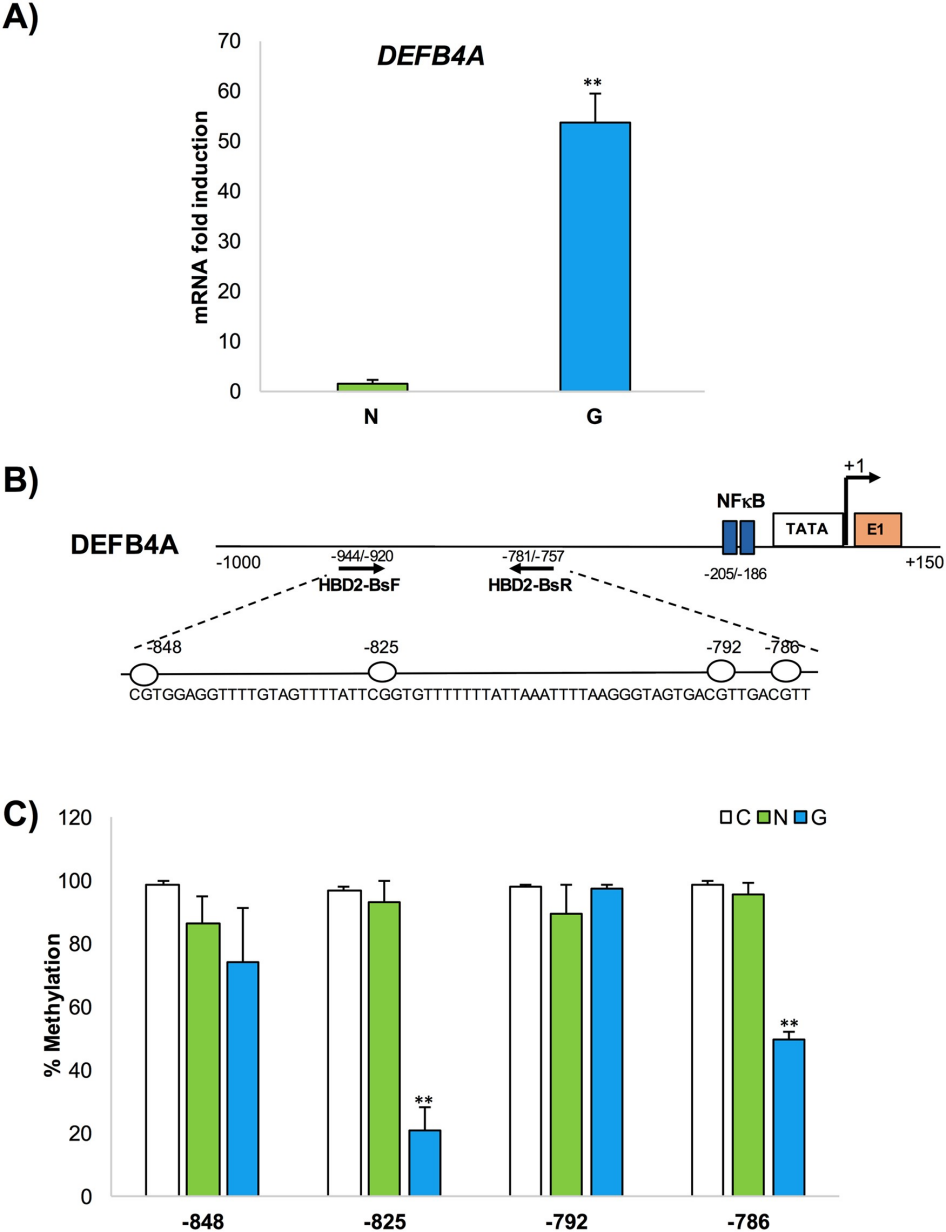
HBD2 gene expression analysis and DNA methylation assay in gastric mucosal tissues. mRNA and genomic DNA was extracted from 15 *H*. *pylori*-negative patients (N) and -positive (G) patients. (A) A qPCR assay measured HBD2 mRNA expression. (B) Schematic representation of HBD2 sequence: TTS (+1), NFKB sites and primers positions used for methylation analysis are indicated. The white lollipop represents CpG dinucleotide positions.

Consistent with precedent reports, HBD2 gene expression was 53.6-fold higher in *H*. *pylori*-positive (G) patients than in *H*. *pylori*-negative (N) patients (Fig 1A). Moreover, we have also examined the HBD1 (gene name *DEFB1)* expression levels in the same gastric biopsy samples and we found that HBD1 transcripts do not differ significantly between *H*. *pylori*-negative and positive patients. This is consistent with most previous studies in this field [41, 46, 47] (S1 Fig).

Methylation analysis of the HBD2 promoter regions was performed on genomic DNA extracted from human tissue gastric samples in 15 controls and 10 patients colonized by *H*. *pylori*, obtained from surgical specimens. A diagram of the *HBD2* gene, including the relative positions of the analyzed CpG sites, is shown in Fig 1B. To examine the level of methylation of HBD2 promoter region, a DNA methylation analysis was performed. We analyzed an HBD2 promoter region with 4 CpG sites at nucleotide positions -786, -792, -825, and -848 with respect to the transcription start site (TSS) (Fig 1B). The primers used for DNA methylation analysis, targeting the region from -944 to -757, showed the HBD2 methylation degree for each CpG site as indicated in a histogram of a tissue sample (Fig 1C). Marked differential methylation was detected between the *H*. *pylori*-positive gastritis (G) and *H*. *pylori*-negative (N) patients. A high methylation degree (up to 85%) at the 4 CpG sites was observed in all *H*. *pylori*-negative samples, while a decrease in methylation degree (20–50%) was observed in at least 2 of the 4 CpG sites in the *H*. *pylori*-positive gastritis. The specific demethylation observed in two CpG sites (-825 and -786) on the HBD2 promoter region could be related to the active transcription pattern of the gene in *H*. *pylori*-positive gastritis (Fig 1A).

### Identification of putative DEFB4B (HBD2) transcription factor binding sites

Since DNA demethylation of HBD2 promoter region is associated with gene expression, we wondered if the region between -825 and -786 nucleotides contains specific transcription factor binding sites (TFBS). To investigate whether this sequence includes some transcription factor binding sites, we performed sequence analysis with the TFBIND program [48]. TFBIND estimates transcription factor DNA binding ability. Putative transcription factor binding sites are indicated in Table 3. These results reveal that the examined genomic DNA fragment containing CpG sites could be involved in a chromatin conformation change induced by specific DNA demethylation in a region including transcription factor binding sites like NFKB, GATA1, or HSF1 (Table 3).

**Table 3:** Identification of DEFB4A transcription factor binding sites using TFBIND.

ID*1	Score*2	Strand*3	Consensus sequence*4	Identified sequence*5
**V$GATA1_04**	0.820159	+	NNCWGATARNNNN	AGCAGAGAAAGCC
**V$NFKAPPAB_01**	0.784270	+	GGGAMTTYCC	GAGAAAGCCC
**V$P53_02**	0.791032	-	NGRCWTGYCY	GAGAAAGCCC
**V$SP1_01**	0.844281	-	GRGGCRGGGW	AGAAAGCCCT
**V$NFKAPPAB_01**	0.786267	-	GGGAMTTYCC	AGAAAGCCCT
**V$HSF1_01**	0.795195	+	RGAANRTTCN	AGAAAGCCCT
**V$GC_01**	0.822391	-	NRGGGGCGGGGCNK	AAGCCCTGGCTCCC
**V$SP1_Q6**	0.762756	-	NGGGGGCGGGGYN	AGCCCTGGCTCCC
**V$SP1_01**	0.801246	-	GRGGCRGGGW	GCCCTGGCTC
**V$AHRARNT_02**	0.775531	-	GRGKATYGCGTGMSWNSCC	GCCCTGGCTCCCAAAGCCC
**V$ER_Q6**	0.740494	-	NNARGNNANNNTGACCYNN	CTGGCTCCCAAAGCCCTGA
**V$IK2_01**	0.854058	-	NNNYGGGAWNNN	TGGCTCCCAAAG
**V$GRE_C**	0.802055	+	GGTACAANNTGTYCTK	GGCTCCCAAAGCCCTG
**V$E2F_02**	0.743405	-	TTTSGCGC	GCTCCCAA
**V$LYF1_01**	0.897266	-	TTTGGGAGR	GCTCCCAAA
**V$AP2_Q6**	0.875230	+	MKCCCSCNGGCG	CTCCCAAAGCCC
**V$CETS1P54_01**	0.836230	+	NCMGGAWGYN	CCCTGAAGTC
**V$NRF2_01**	0.824128	+	ACCGGAAGNS	CCCTGAAGTC
**V$CREB_01**	0.789243	+	TGACGTMA	TGAAGTCC

*^1^ ID indicates transcription factor matrix from the transcription factor database TRANSFAC R. 3.3.

*^2^ Score shows similarity (0.0–1.0) between a registered sequence for the transcription factor binding sites and the identified sequence.

*^3^ DNA strand.

*^4^ Consensus sequence of the transcription factor binding sites. S = C or G, W = A or T, R = A or G, Y = C or T, K = G or T, M = A or C, N = any base pair.

*^5^ Sequence identified by TFBIND from the input sequence (DEFB4A sequence analyzed: from -825 to -786 nucleotides).

### HBD3-4 expression and DNA methylation analysis in *H*. *pylori*-positive patients

**Fig 2:**
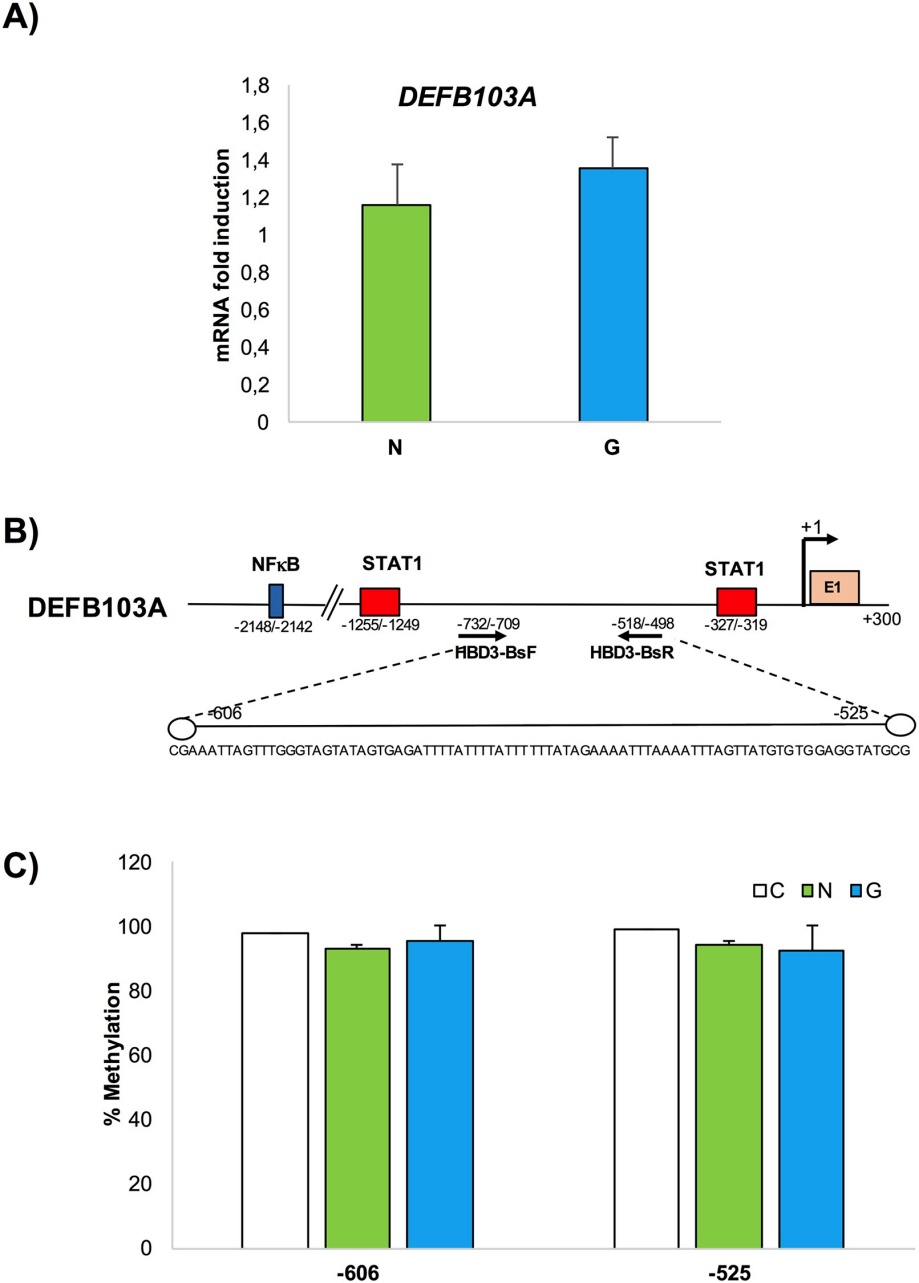
HBD3 gene expression analysis and DNA methylation assay in gastric mucosal tissues. mRNA and genomic DNA was extracted from 15 *H*. *pylori*-negative patients (N) and -positive (G) patients. (A) qPCR measured HBD3 mRNA expression. (**B)** Schematic representation of HBD3 sequence: TTS (+1), STAT1, NFKB sites, and primer positions used for methylation analysis are indicated. The white lollipop represents CpG dinucleotide positions.

**Fig 3:**
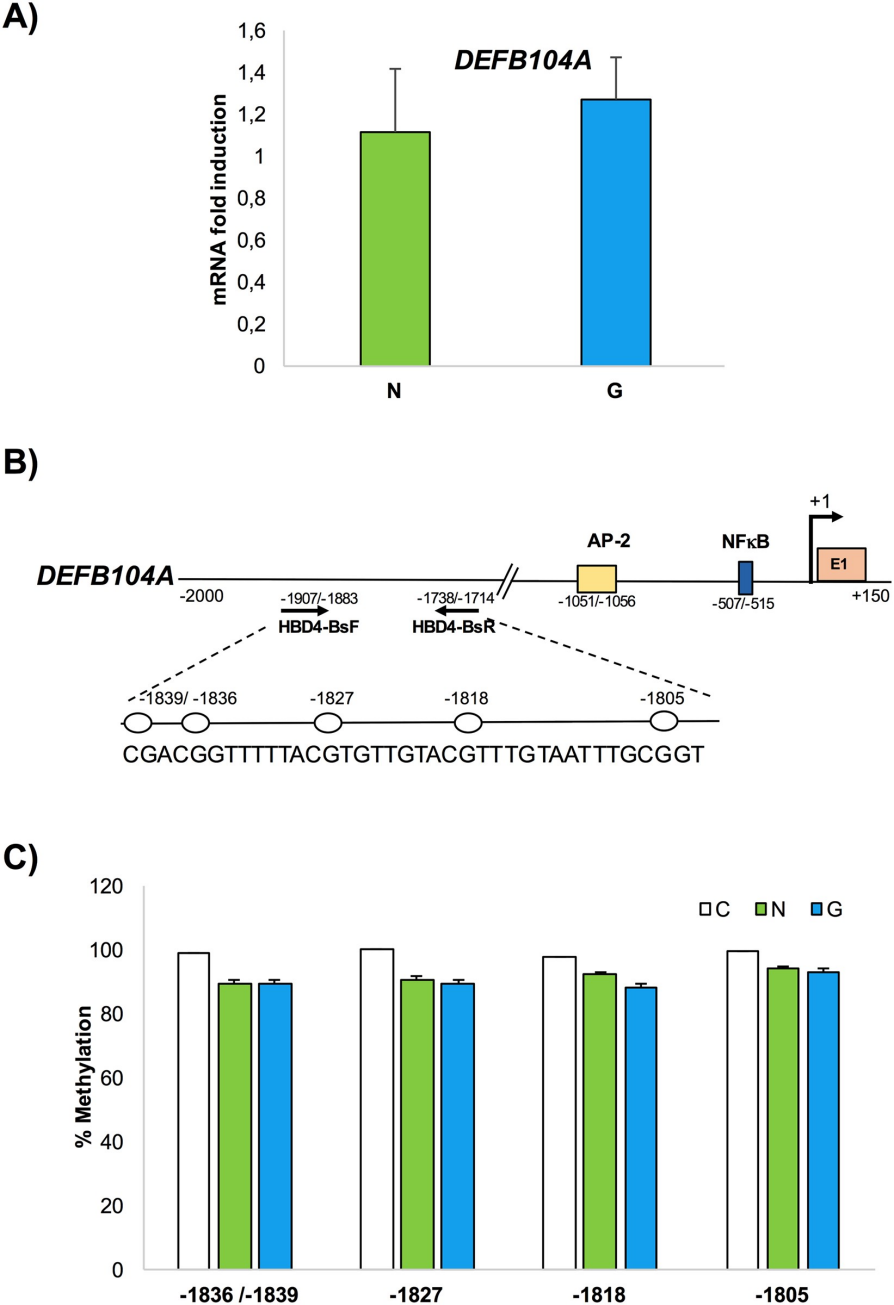
HBD4 gene expression analysis and DNA methylation assay in gastric mucosal tissues. mRNA and genomic DNA was extracted from 15 *H*. *pylori*-negative patients (N) and positive (G) patients. **(A)** qPCR measured HBD4 mRNA expression. (**B)** Schematic representation of HBD4 sequence: TTS (+1), AP-2, NFKB sites, and primer positions used for methylation analysis are indicated. The white lollipop represents CpG dinucleotide positions.

In contrast to gene expression levels observed for HBD2 in *H*. *pylori*-positive patients, HBD3 and HBD4 gene expression was only marginally detected in the human gastric mucosa independent of *H*. *pylori* infection (Figs 2A and 3A).

Taken together, these data indicate that HBD2 gene expression was induced in *H*. *pylori* positive samples by inflammation. In contrast, HBD3 and HBD4 expression levels were barely detected. Even for the HBD3 and HBD4 genes we evaluated the methylation state of 2 (nucleotide positions -606, and -525) and 4 CpG sites (nucleotide positions -1836/1839, -1827, -1818, and -1805) targeting the regions from -732 to -498 (Fig 2B) and from -1907 to -1714 (Fig 3B), respectively. The results were plotted on a histogram displaying the methylation degree of each CpG site in each tissue sample (Figs 2C and 3C). For both genes HBD3 and HBD4, no differential methylation was observed between *H*. *pylori*-positive gastritis and *H*. *pylori*-negative patients in all analyzed CpG sites.

The results of triplicate experiments indicated differential methylation in *H*. *pylori*-positive gastritis and *H*. *pylori* negative patients only in the HBD2 genomic region.

#### Antibacterial activities of the 3NI analog and wild-type HBDs

Antimicrobial activity of HBD2-4 and the analogs 3N and 3NI was evaluated on *H*. *pylori*, cultured on HP agar for 4 d after a 1-h pre-incubation with or without chemically synthesized HBD-2-4 and 3N and 3NI analogs. The two peptide sequences are derived from HBD1 and HBD3. 3N analog is the HBD3 sequence without the N-terminal domain that HBD1 lacks. As for 3NI, this sequence is the most active and corresponds to 3N in which the inner HBD1 domain is responsible of the antimicrobial activity [36] substitutes the same domain of HBD3.

**Fig 4:**
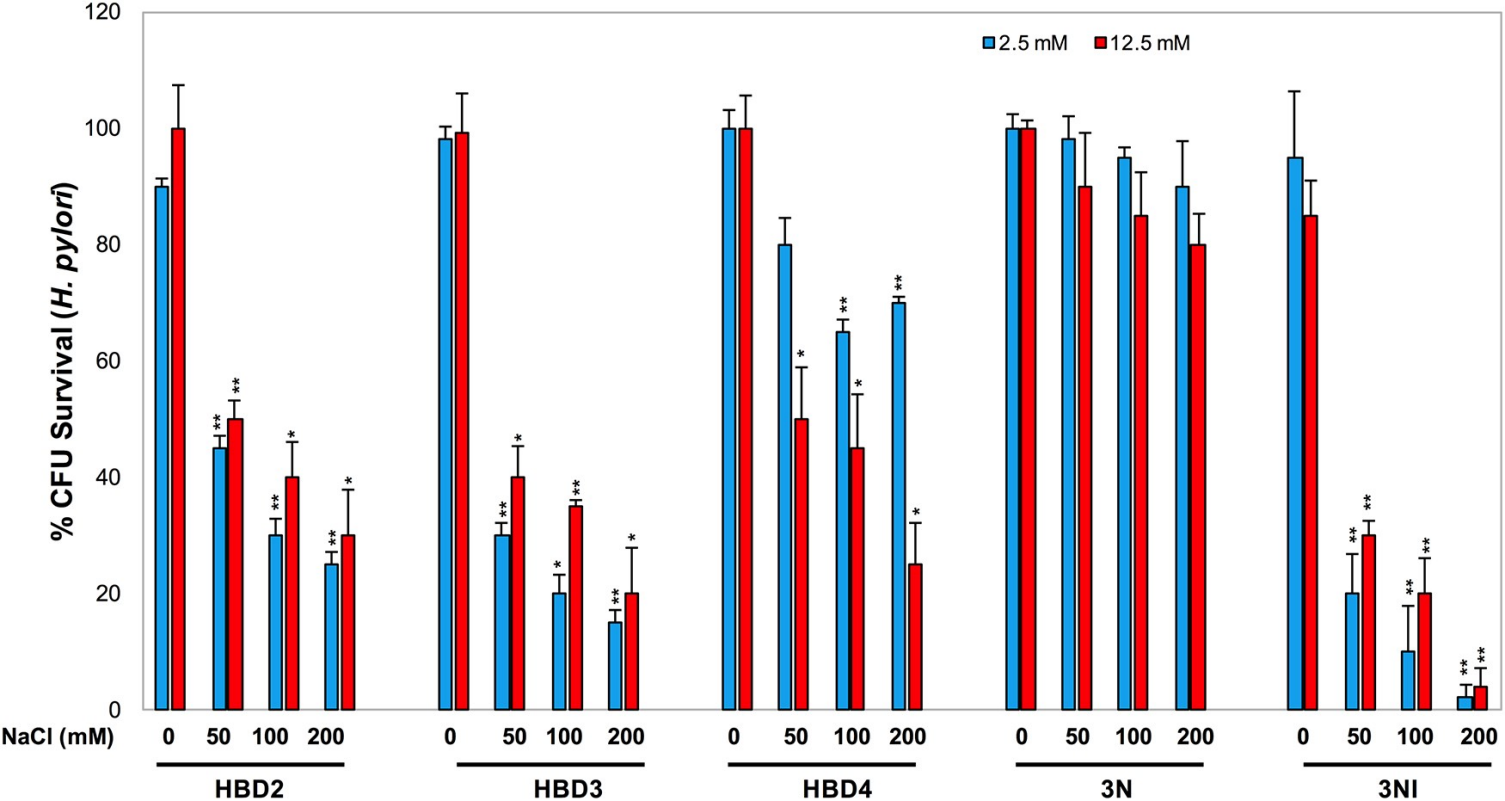
The 3NI analog has increased antibacterial activity against *H*. *pylori* in contrast to wild-type peptides. The antimicrobial activities of wild-type HBD2, HBD3, and HBD4 and the analogs 3N and 3NI were evaluated at two concentrations (2.5 and 12.5 μM) against *H*. *pylori* with 0, 50, 100, and 200 mM NaCl. Error bars show standard deviations (SDs) from 3 independent experiments. Statistical analysis was performed concerning salt point 0 at both two concentrations.

We evaluated two concentrations of every peptide (2.5 and 12.5 μM) and 4 NaCl concentrations (0, 50, 100, and 200 mM). Antibacterial activity of 3NI against *H*. *pylori* started at concentrations as low as 2.5 μM in the presence of at least 200 mM NaCl. The MIC values were between 12.5 and 25.0 μM. Each peptide exerted strong antibacterial activity at 12.5 μM against *H*. *pylori* except for the analog 3N, which showed little antibacterial effect at all four tested NaCl concentrations (Fig 4).

## Discussion

The stomach protects against microbes using the low gastric pH and the secretion of antimicrobial peptides and mucins by epithelial cells. *H*. *pylori* gastritis correlates with up-regulation of various antimicrobials. For instance, the amounts of HBD2 but not HBD1 are increased in gastric mucosae of *H*. *pylori*-positive specimens [49,50]. Here, we compared the expression of HBD2-4 genes in the gastric mucosa with or without *H*. *pylori*, and we compared these changes to anti-*H*. *pylori* activity. Moreover, we analyzed the development of an epigenetic basis for cancer formation from HBD2 during *H*. *pylori*-associated gastric tumorigenesis. qPCR analysis of hBD2-4 gene expression revealed that expression powerfully increased HBD2 levels in *H*. *pylori*-infected gastric mucosa compared to uninfected, and this agreed with previously published data [32,39–42,46,47,51]. Interestingly there was no up-regulation of HBD3 and HBD4. It has been suggested that HBD3 can be induced by *H*. *pylori*, but HBD3 was unaltered in the gastric antrum and imperceptible in biopsies of corpus in uninfected children [52–54]. Therefore, it is possible that previous studies reporting an increase of HBD3 gene expression in *H*. *pylori* gastritis are somewhat irrelevant in quantitative terms.

In addition, *H pylori* may induce the expression of HBD3 by activating NF-κB or other signaling pathways. Kawauchi et al., [41] found that anti-TLR-4 antibody partially repressed HBD3 expression in MKN45 cells after *H*. *pylori* induction. This suggests that different functions may be responsible for *H*. *pylori* infection-dependent HBD3 induction in gastric cells. Moreover, HBD3 mRNA expression levels did not relate with HBD2 in *H*. *pylori*-positive specimens. In addition, this also indicated that HBD3 and HBD4 mRNA expression in gastric epithelial cells is probably controlled via another mechanism.

In contrast to other studies [47,55] we observed no prominent HBD4 gene expression in the stomach. It is still unknown whether *H*. *pylori* itself or the inflammatory process-linked cytokines are responsible for HBD2 induction [47,56]. On the one hand, experiments in various gastric adenocarcinoma cell lines after *H*. *pylori* infection showed increased HBD2 and HBD3 expression [39,52,31,57]. On the other hand, induction with IL-1β also led to increased HBD2 expression [39].

Finally, we tested the *H*. *pylori tox+* strain 60190 susceptibility toward the antimicrobial peptides of mucosal surfaces (HBD2-4 and analogs 3N and 3NI). In our experiments, the inducible defensins HBD2-HBD4 showed a robust antibacterial effect towards *H*. *pylori*.

DNA methylation analysis demonstrated that the methylation status of the HBD2 gene promoter was higher in *H*. *pylori-*negative chronic gastritis than *H*. *pylori-*positive chronic gastritis. Among the human beta-defensins (HBD2-4) analyzed, the grade of methylation was modulated only in HBD2. Surprisingly, we observed a correlation with HBD2 gene expression even if only 2 sites (-825 and -786) are demethylated at the HBD2 promoter. qRT-PCR analysis showed significant up-regulation of HBD-2 gene expression between *H*. *pylori*-negative chronic gastritis to *H*. *pylori*-positive chronic gastritis. It is well described that CpG methylation has an effect on native chromatin status, and this connection is modulated by methyl-binding proteins that preserve the capacity to engage chromatin repressor complexes on methylated DNA. In our previous work, we showed that the demethylation of a specific CpG site at the RET region enhancer promotes the shift of the methyl binding protein MeCP2, inducing transcriptional reactivation of the gene [58]. Moreover, it was observed that the demethylation of a single specific CpG site is required for *hIL2* (human interleukin 2 gene) transcription, and the epigenetic marker formed constitutes a memory of the regulatory event [59]. A similar result was obtained after single CpG demethylation of the L2RA locus gene, a subunit of the high affinity receptor for interleukin-2 (IL2). L2RA interprets a critical function in immune homeostasis. In fact, its single demethylation site corresponds to its expression in CD4+ T cells [60]. Finally, prominent demethylation of a single cytosine residue situated in the 5th exon of the PGLYRP3 gene is associated with an increment of mRNA level of PGLYRP3 [61]. This information supports our hypothesis that a single demethylation in one specific CpG dinucleotide could reactivate the gene.

We did not reveal a divergence in the DNA methylation and mRNA expression levels of HBD3 and HBD4 in chronic gastritis with and without *H*. *pylori* infection. The gap could be due to the failure of quantification molecules that have aberrant DNA methylation. An abnormal degree of methylation in non-cancerous tissues happens only in a percentage of cells, which is predicted to be highly variable. Qualitative analysis of methylation does not seem appropriate. Also, several CpG islands (CGI) and, even inside a CGI, diverse regions show different susceptibilities to aberrant DNA methylation [62], and analysis considering the different susceptibilities has not been done. More importantly, there are no studies on the relationship between aberrant DNA methylation in the gastric mucosa and the risks of gastric cancer. It appears that modifications of DNA methylation could interact with *H*. *pylori* infection and other carcinogen agents and that these methylation alterations could affect cancer risk.

It has been suggested that this variance might occur because aberrant methylation develops in only a percentage of cells. Also, DNA methylation is intimately associated with metaplasia of the gastric mucosa. For example, hypermethylation of APC, THBD, and HAND1 was associated with gastric metaplasia [63]. Our findings may be interpreted as HBD2 epigenetic silencing occurring after GC generation rather than before the spread of cancer or during the formation of the epigenetic field. Another possible interpretation could be that an epigenetic action different from DNA methylation, like histone modification, is involved in increased mRNA expression [64].

## Conclusion

Our study suggests that *H*. *pylori* can induce HBD2 expression and modulation of DNA methylation. It is possible that HBD2 induction minimizes competition by other more susceptible bacteria. Besides, *H*. *pylori* does not induce peptides like HBD3 and HBD4. The association of defensin elicitation and resistance to other bacteria may enable *H*. *pylori* to colonize the gastric mucosa where it can join epithelial cells and induce inflammatory and cancerous development.

Our study provides the first comprehensive analysis of transcript and DNA methylation levels of one of the most prominent human beta-defensins in patients infected with *H*. *pylori*.

A finer insight of the mechanisms regarding resistance and susceptibility of *H*. *pylori* against other antimicrobial peptides might favor the detection of possible targets for new eradication therapeutics.

## Supporting information

S1 Fig *DEFB1* gene expression analysis in gastric mucosal tissues. mRNA was extracted from 15 *H*. *pylori*-negative patients (N) and 10 -positive (G) patients. *DEFB1B* mRNA expression was measured using qPCR.
(TIFF)

## References

[1] Itzkowitz SH, Yio X. Inflammation and cancer IV. Colorectal cancer in inflammatory bowel disease: the role of inflammation. Am J Physiol Gastrointest Liver Physiol. 2004; 287: G7–17. 10.1152/ajpgi.00079.2004 15194558

[2] Coretti L, Natale A, Cuomo M, Florio E, Keller S, Lembo F, et al The Interplay between Defensins and Microbiota in Crohn’s Disease. Mediators Inflamm. 2017; 2017: 8392523 10.1155/2017/8392523 28246439 PMC5299173

[3] Stauffer JK, Scarzello A, Jiang Q, Wiltrout RH. Chronic inflammation, immune escape and oncogenesis in the liver: a unique neighbourhood for novel intersections. Hepatology. 2012; 56: 1567–74. 10.1002/hep.25674 22378061 PMC3381981

[4] Macarthur M., Hold GL, El-Omar EM. Inflammation and Cancer II. Role of chronic inflammation and cytokine gene polymorphisms in the pathogenesis of gastrointestinal malignancy. Am J Physiol Gastrointest Liver Physiol. 2004; 286: G515–G520. 10.1152/ajpgi.00475.2003 15010360

[5] Lu H, Ouyang W, Huang C. Inflammation, a key event in cancer development. Mol Cancer Res. 2006; 4: 221–233. 10.1158/1541-7786.MCR-05-0261 16603636

[6] Chiariotti L, Coretti L, Pero R, Lembo F. Epigenetic Alterations Induced by Bacterial Lipopolysaccharides. Adv Exp Med Biol. 2016; 879: 91–105. 10.1007/978-3-319-24738-0_5 26659265

[7] Rutter M, Saunders B, Wilkinson K, Rumbles S, Schofield G, Kamm M, et al Severity of inflammation is a risk factor for colorectal neoplasia in ulcerative colitis. Gastroenterology. 2004; 126: 451–459. 10.1053/j.gastro.2003.11.010 14762782

[8] Chen CJ, Yang HI, Su J, Jen CL, You SL, Lu SN, et al Risk of hepatocellular carcinoma across a biological gradient of serum hepatitis B virus DNA level. JAMA. 2006; 295: 65–73. 10.1001/jama.295.1.65 16391218

[9] Yang HI, Yeh SH, Chen PJ, Iloeje UH, Jen CL, Su J, et al Associations between hepatitis B virus genotype and mutants and the risk of hepatocellular carcinoma. J Natl Cancer Inst. 2008; 100: 1134–1143. 10.1093/jnci/djn243 18695135 PMC2518166

[10] Correa P, Houghton J. Carcinogenesis of *Helicobacter pylori*. Gastroenterology. 2007; 133: 659–667. 10.1053/j.gastro.2007.06.026 17681184

[11] Pero R, Coretti L, Nigro E, Lembo F, Laneri S, Lombardo B, et al β-Defensins in the Fight against Helicobacter pylori. Molecules. 2017; 7: 3. 10.3390/molecules22030424 PMC6155297 28272373

[12] Feinberg AP, Ohlsson R, Henikoff S. The epigenetic progenitor origin of human cancer. Nat Rev Genet. 2006; 7: 21–33. 10.1038/nrg1748 16369569

[13] Ushijima T. Epigenetic field for cancerization. J Biochem Mol Biol. 2007; 40: 142–150. 17394762 10.5483/bmbrep.2007.40.2.142

[14] Niwa T, Tsukamoto T, Toyoda T, Mori A, Tanaka H, Maekita T, et al Inflammatory processes triggered by *Helicobacter pylori* infection cause aberrant DNA methylation in gastric epithelial cells. Cancer Res. 2010; 70: 1430–1440. 10.1158/0008-5472.CAN-09-2755 20124475

[15] Maekita T, Nakazawa K, Mihara M, Nakajima T, Yanaoka K, Iguchi M, et al High levels of aberrant DNA methylation in *Helicobacter pylori* infected gastric mucosae and its possible association with gastric cancer risk. Clin Cancer Res. 2006; 12: 989–995. 10.1158/1078-0432.CCR-05-2096 16467114

[16] Enomoto S, Maekita T, Tsukamoto T, Nakajima T, Nakazawa K, Tatematsu M, et al Lack of association between CpG island methylator phenotype in human gastric cancers and methylation in their background non-cancerous gastric mucosae. Cancer Sci. 2007; 98: 1853–1861. 10.1111/j.1349-7006.2007.00625.x 17900260 PMC11158991

[17] Kondo Y, Kanai Y, Sakamoto M, Mizokami M, Ueda R, Hirohashi S. Genetic instability and aberrant DNA methylation in chronic hepatitis and cirrhosis: a comprehensive study of loss of heterozygosity and microsatellite instability at 39 loci and DNA hypermethylation on 8 CpG islands in micro dissected specimens from patients with hepatocellular carcinoma. Hepatology. 2000; 32: 970–979. 10.1053/jhep.2000.19797 11050047

[18] Issa JP, Ahuja N, Toyota M, Bronner MP, Brentnall TA. Accelerated age-related CpG island methylation in ulcerative colitis. Cancer Res. 2001; 61: 3573–3577. 11325821

[19] Chiariotti L, Angrisano T, Keller S, Florio E, Affinito O, Pallante P, et al Epigenetic modifications induced by *Helicobacter pylori* infection through a direct microbe-gastric epithelial cells cross-talk. Med Microbiol Immunol. 2013; 202: 327–337. 10.1007/s00430-013-0301-6 23715627

[20] Ohata H, Kitauchi S, Yoshimura N, Mugitani K, Iwane M, Nakamura H, et al Progression of chronic atrophic gastritis associated with *Helicobacter pylori* infection increases risk of gastric cancer. Int J Cancer. 2004; 109: 138–143. 10.1002/ijc.11680 14735480

[21] Yakirevich E, Resnick MB. Pathology of gastric cancer and its precursor lesions. Gastroenterol Clin North Am. 2013; 42: 261–284. 10.1016/j.gtc.2013.01.004 23639640

[22] Gigek CO, Chen ES, Calcagno DQ, Wisnieski F, Burbano R, Smith MA. Epigenetic mechanisms in gastric cancer. Epigenomics. 2012; 4: 279–294. 10.2217/epi.12.22 22690664

[23] Zhao CX. Promoter methylation of tumor-related genes in gastric carcinogenesis. Histol Histopathol. 2012; 27: 1271–1282. 10.14670/HH-27.1271 22936446

[24] Chan AO, Lam SK, Wong BC, Wong WM, Yuen MF, Yeung YH, et al Promoter methylation of E-cadherin gene in gastric mucosa associated with *Helicobacter pylori* infection and in gastric cancer. Gut. 2003; 52: 502–506. 10.1136/gut.52.4.502 12631658 PMC1773595

[25] Chan AO, Rashid A. CpG island methylation in precursors of gastrointestinal malignancies. Curr Mol Med. 2006; 6: 401–408. 16900663 10.2174/156652406777435417

[26] Nigro E, Colavita I, Sarnataro D, Scudiero O, Zambrano G, Granata V, et al An ancestral host defence peptide within human β-defensin recapitulates the antibacterial and antiviral activity of the full-length molecule. Sci Rep. 2015; 21: 5–18450. 10.1038/srep18450 PMC4685272 26688341

[27] Boughan PK, Argent RH, Body-Malapel M, Park JH, Ewings KE, Bowie AG, et al Nucleotide-binding oligomerization domain-1 and epidermal growth factor receptor: critical regulators of beta-defensins during *Helicobacter pylori* infection. J Biol Chem. 2006; 281: 11637–11648. 10.1074/jbc.M510275200 16513653

[28] Grubman A, Kaparakis M, Viala J, Allison C, Badea L, Karrar A, et al The innate immune molecule, NOD1, regulates direct killing of *Helicobacter pylori* by antimicrobial peptides. Cell Microbiol. 2010; 12: 626–639. 10.1111/j.1462-5822.2009.01421.x 20039881

[29] Bauer B, Wex T, Kuester D, Meyer T, Malfertheiner P. Differential expression of human beta defensin 2 and 3 in gastric mucosa of *Helicobacter pylori*-infected individuals. *Helicobacter*. 2013; 18: 6–12. 10.1111/hel.12000 23067102

[30] Muhammad JS, Zaidi SF, Zhou Y, Sakurai H, Sugiyama T. Novel epidermal growth factor receptor pathway mediates release of human β-defensin 3 from *Helicobacter pylori*-infected gastric epithelial cells. Pathog Dis. 2016; 74. 10.1093/femspd/ftv128 26733497

[31] O’Neil DA. Regulation of expression of beta-defensins: endogenous enteric peptide antibiotics. Mol Immunol. 2003; 40: 445–450. 10.1016/s0161-5890(03)00161-5 14568390

[32] Taha AS, Faccenda E, Angerson WJ, Balsitis M, Kelly RW. Gastric epithelial anti-microbial peptides-histological correlation and influence of anatomical site and peptic ulcer disease. Dig Liver Dis. 2005; 37: 51–56. 10.1016/j.dld.2004.07.019 15702860

[33] Patel SR, Smith K, Letley DP, Cook KW, Memon AA, Ingram RJ, et al *Helicobacter pylori* downregulates expression of human β-defensin 1 in the gastric mucosa in a type IV secretion-dependent fashion. Cell Microbiol. 2013; 15: 2080–2092. 10.1111/cmi.12174 23870035 PMC4028989

[34] Selsted ME, Ouellette AJ. Mammalian defensins in the antimicrobial immune response. Nat Immunol. 2005; 6: 551–557. 10.1038/ni1206 15908936

[35] Scudiero O, Galdiero S, Cantisani M, Di Noto R, Vitiello M, Galdiero M, et al Novel synthetic, salt-resistant analogs of human beta-defensins 1 and 3 endowed with enhanced antimicrobial activity. Antimicrob Agents Chemother. 2010; 54: 2312–2322. 10.1128/AAC.01550-09 20308372 PMC2876405

[36] Scudiero O, Galdiero S, Nigro E, Del Vecchio L, Di Noto R, Cantisani M. et al Chimeric beta-defensin analogs, including the novel 3NI analog, display salt-resistant antimicrobial activity and lack toxicity in human epithelial cell lines. Antimicrob Agents Chemother. 2013; 57: 1701–1708. 10.1128/AAC.00934-12 23357761 PMC3623310

[37] Lombardi L, Shi Y, Falanga A, Galdiero E, De Alteriis E, Franci G et al Enhancing the potency of antimicrobial peptides through molecular engineering and self-assembly. Biomacromolecules. 2019; 20: 1362–1374. 10.1021/acs.biomac.8b01740 30735368

[38] Scudiero O, Nigro E, Cantisani M, Colavita I, Leone M, Mercurio FA, et al Design and activity of a cyclic mini-β-defensin analog: a novel antimicrobial tool. Int J Nanomedicine. 2015; 10: 6523–6539. 10.2147/IJN.S89610 26508857 PMC4610797

[39] Bajaj-Elliott M, Fedeli P, Smith GV, Domizio P, Maher L, Ali RS, et al Modulation of host antimicrobial peptide (beta-defensins 1 and 2) expression during gastritis. Gut. 2002; 51: 356–361. 10.1136/gut.51.3.356 12171956 PMC1773366

[40] Hamanaka Y, Nakashima M, Wada A, Ito M, Kurazono H, Hojo H, et al Expression of human beta-defensin 2 (hBD-2) in *Helicobacter pylori* induced gastritis: antibacterial effect of hBD-2 against *Helicobacter pylori*. Gut. 2001; 49; 481–487. 10.1136/gut.49.4.481 11559643 PMC1728463

[41] Wehkamp J, Schmidt K, Herrlinger KR, Baxmann S, Behling S, Wohlschläger C, et al Defensin pattern in chronic gastritis: HBD-2 is differentially expressed with respect to *Helicobacter pylori* status. J Clin Pathol. 2003; 56: 352–357. 10.1136/jcp.56.5.352 12719455 PMC1769951

[42] Kawauchi K, Yagihashi A, Tsuji N, Uehara N, Furuya D, Kobayashi D, et al Human beta-defensin-3 induction in *H*. *pylori*-infected gastric mucosal tissues. World J Gastroenterol. 2006; 12: 5793–5797. 10.3748/wjg.v12.i36.5793 17007044 PMC4100659

[43] di Martino O, Troiano A, Guarino AM, Pollice A, Vivo M, La Mantia G, et al ΔNp63α controls YB-1 protein stability: evidence on YB-1 as a new player in keratinocyte differentiation. Genes Cells. 2016; 21: 648–660. 10.1111/gtc.12373 27168020

[44] Keller S, Angrisano T, Florio E, Pero R, Decaussin-Petrucci M, Troncone G et al DNA methylation state of the galectin-3 gene represents a potential new marker of thyroid malignancy. Oncol Lett. 2013; 6: 86–90. 10.3892/ol.2013.1312 23946782 PMC3742793

[45] Angrisano T, Pero R, Brancaccio M, Coretti L, Florio E, Pezone A, et al Cyclical DNA Methylation and Histone Changes Are Induced by LPS to Activate COX-2 in Human Intestinal Epithelial Cells. PLoS. One. 2016; 11: e0156671 10.1371/journal.pone.0156671 27253528 PMC4890762

[46] Hase K, Murakami M, Iimura M, Cole SP, Horibe Y, Ohtake T, et al Expression of LL-37 by human gastric epithelial cells as a potential host defense mechanism against *Helicobacter pylori*. Gastroenterology. 2003; 125: 1613–1625. 10.1053/j.gastro.2003.08.028 14724813

[47] Otte JM, Neumann HM, Brand S, Schrader H, Schmidt WE, Schmitz F. Expression of beta-defensin 4 is increased in human gastritis. Eur J Clin Invest. 2009; 39: 126–128. 10.1111/j.1365-2362.2008.02071.x 19200166

[48] Tsunoda T, Takagi T. Estimating transcription factor bind ability on DNA. Bioinformatics. 1999; 815: 622–630. 10.1093/bioinformatics/15.7.622 10487870

[49] Isomoto H, Mukae H, Ishimoto H, Nishi Y, Wen CY, Wada A, et al High concentrations of human beta-defensin 2 in gastric juice of patients with *Helicobacter pylori* infection. World J Gastroenterol. 2005; 11: 4782–4787. 10.3748/wjg.v11.i31.4782 16097044 PMC4398722

[50] Nishi Y, Isomoto H, Mukae H, Ishimoto H, Wen CY, Wada A, et al Concentrations of alpha- and beta-defensins in gastric juice of patients with various gastroduodenal diseases. World J Gastroenterol. 2005; 11: 99–103. 10.3748/wjg.v11.i1.99 15609405 PMC4205393

[51] Uehara N, Yagihashi A, Kondoh K, Tsuji N, Fujita T, Hamada H, et al Human beta-defensin-2 induction in *Helicobacter pylori*-infected gastric mucosal tissues: antimicrobial effect of overexpression. J Med Microbiol. 2003; 52: 41–45. 10.1099/jmm.0.04985-0 12488564

[52] Vordenbäumen S, Pilic D, Otte JM, Schmitz F, Schmidt-Choudhury A. Defensin-mRNA expression in the upper gastrointestinal tract is modulated in children with celiac disease and *Helicobacter pylori* positive gastritis. J Pediatr Gastroenterol Nutr. 2010; 50: 596–600. 10.1097/MPG.0b013e3181cd26cd 20400909

[53] Islam D, Bandholtz L, Nilsson J, Wigzel H, Christensson B, Agerberth B, et al Downregulation of bactericidal peptides in enteric infections: a novel immune escape mechanism with bacterial DNA as a potential regulator. Nat Med. 2001; 7: 180–185. 10.1038/84627 11175848

[54] Hosaka Y, Koslowski M, Nuding S, Wang G, Schlee M, Schäfer C, et al Antimicrobial host defense in the upper gastrointestinal tract. Eur J Gastroenterol Hepatol. 2008; 20: 1151–1158. 10.1097/MEG.0b013e3283052ddb 18989140

[55] Resnick MB, Sabo E, Meitner PA, Kim SS, Cho Y, Kim HK, et al Global analysis of the human gastric epithelial transcriptome altered by *Helicobacter pylori* eradication in vivo. Gut. 2006; 55: 1717–1724. 10.1136/gut.2006.095646 16641130 PMC1856477

[56] Ohara T, Morishita T, Suzuki H, Masaoka T, Nishizawa T, Hibi T. Investigation of the possibility of human-beta defensin 2 (hBD2) as a molecular marker of gastric mucosal inflammation. Hepatogastroenterology. 2005; 52: 1320–1324. 16201065

[57] Wada A, Mori N, Oishi K, Hojo H, Nakahara Y, Hamanaka Y, et al Induction of human beta-defensin-2 mRNA expression by *Helicobacter pylori* in human gastric cell line MKN45 cells on cag pathogenicity island. Biochem Biophys Res Commun. 1999; 263: 770–774. 10.1006/bbrc.1999.1452 10512755

[58] Angrisano T, Sacchetti S, Natale F, Cerrato A, Pero R, Keller S, et al Chromatin and DNA methylation dynamics during retinoic acid-induced RET gene transcriptional activation in neuroblastoma cells. Nucleic Acids Res. 2011; 39:1993–2006. 10.1093/nar/gkq864 20952403 PMC3064803

[59] Murayama A, Sakura K, Nakama M, Yasuzawa-Tanaka K, Fujita E, Tateishi Y, et al specific CpG site demethylation in the human interleukin 2 gene promoter is an epigenetic memory. EMBO J. 2006; 25: 1081–1092. 10.1038/sj.emboj.7601012 16498406 PMC1409718

[60] Belot MP, Castell AL, Le Fur S, Bougnères P. Dynamic demethylation of the IL2RA promoter during in vitro CD4+ T cell activation in association with IL2RA expression. Epigenetics. 2018; 13: 459–472. 10.1080/15592294.2018.1469893 30096258 PMC6140818

[61] Sobiak B, Leśniak W. The Effect of Single CpG Demethylation on the Pattern of DNA-Protein Binding. Int J Mol Sci. 2019; 20: 914. 10.3390/ijms20040914 PMC6413078 30791552

[62] Ushijima T. Detection and interpretation of altered methylation patterns in cancer cells. Nat Rev Cancer. 2005; 5: 223–231. 10.1038/nrc1571 15719030

[63] Shin CM, Kim N, Jung Y, Park JH, Kang GH, Kim JS, et al Role of *Helicobacter pylori* infection in aberrant DNA methylation along multistep gastric carcinogenesis. Cancer Sci. 2010; 101: 1337–1346. 10.1111/j.1349-7006.2010.01535.x 20345486 PMC11159191

[64] Hayashi Y, Tsujii M, Wan J, Kondo J, Akasaka T, Jin Y, et al Cag A mediates epigenetic regulation to attenuate let-7 expression in *Helicobacter pylori*-related carcinogenesis. Gut. 2013; 62: 1536–1546. 10.1136/gutjnl-2011-301625 22936674

