# Supplementary figures and images for: Beta-defensins and analogs in Helicobacter pylori infections: mRNA expression levels, DNA methylation, and antibacterial activity
Source: PLoS One. 2019 Sep 19;14(9):e0222295. doi: 10.1371/journal.pone.0222295 (PMC6752957; doi:10.1371/journal.pone.0222295)

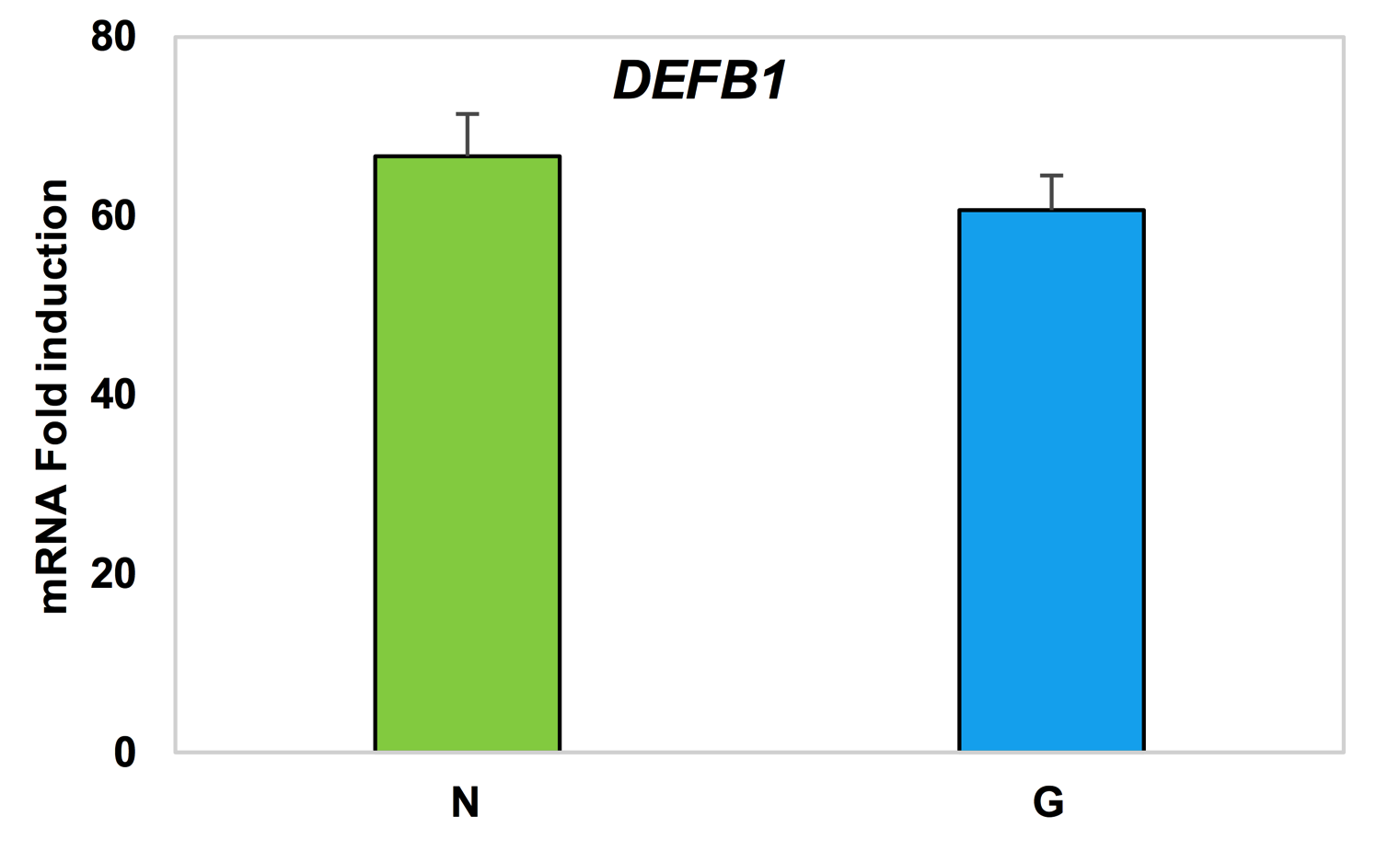

Supplement: S1 Fig — mRNA was extracted from 15 H. pylori-negative patients (N) and 10 -positive (G) patients. DEFB1B mRNA expression was measured using qPCR. (TIFF) [file pone.0222295.s001.tiff]
